# Supplementary material for: Tissue Adhesive, Self-Healing, Biocompatible, Hemostasis, and Antibacterial Properties of Fungal-Derived Carboxymethyl Chitosan-Polydopamine Hydrogels
Source: Pharmaceutics. 2022 May 10;14(5):1028. doi: 10.3390/pharmaceutics14051028 (PMC9145872; doi:10.3390/pharmaceutics14051028)
Supplement: Supplementary file 1 [file pharmaceutics-14-01028-s001.zip › pharmaceutics-1684629-supplementary.pdf]

Supporting Information

Tissue adhesive, self-healing, biocompatible, hemostasis, and antibacterial properties of fungal-derived carboxymethyl chitosan-polydopamine hydrogels

Kummara Madhusudana Rao [1,2], Kannan Badri Narayanan [1,2], Uluvangada Thammaiah [1], Pil-Hoon Park [2,3], Inho Choi[2,4] and Sung Soo Han [1,2,*]

[1] School of Chemical Engineering, Yeungnam University, Gyeongsan 38541, Republic of Korea; msraochem@yu.ac.kr (K.M. Rao); okbadri@gmail.com (K.B.N); sanjuuthappa@gmail.com (U.T.U)
[2] Research Institute of Cell Culture, Yeungnam University, Gyeongsan, Gyeongbuk, 38541, Republic of Korea.
[3] School of Pharmacy, Yeungnam University, Gyeongsan 38541, Republic of Korea; parkp@yu.ac.kr (P.H.P)
[4] Department of Medical Biotechnology, Yeungnam University, Gyeongsan 38541, Republic of Korea; inhochoi@ynu.ac.kr (I.C)
* Correspondence: sshan@yu.ac.kr (S.S.H)

Table S1: Formulations of hydrogels

Formulations	DA/AAm (Wt %)	DA (mg)	FCMCS (g)	AM (g)	BIS (mg)	APS (mg)	TEMED (μL)
FCMCS	0	0	0.25	2	5.0	50	10
0.2DA-FCMCS	0.2	4	0.25	2	5.0	200	10
0.4DA-FCMCS	0.4	8	0.25	2	5.0	200	10

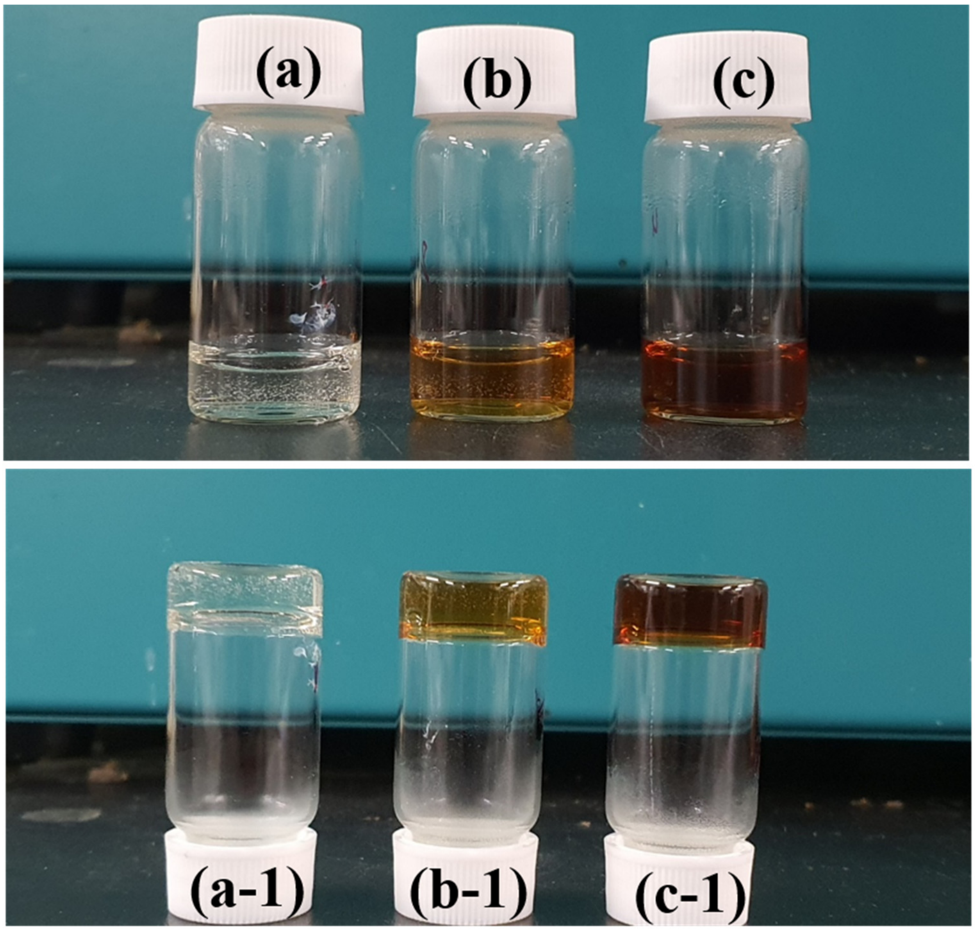

Figure S1. Digital photographs of hydrogel formulations (a) and (a-1) FCMCS (b) and (b-1) 0.2DA-FCMCS, (c) and (c-1) 0.4DA-FCMCS.

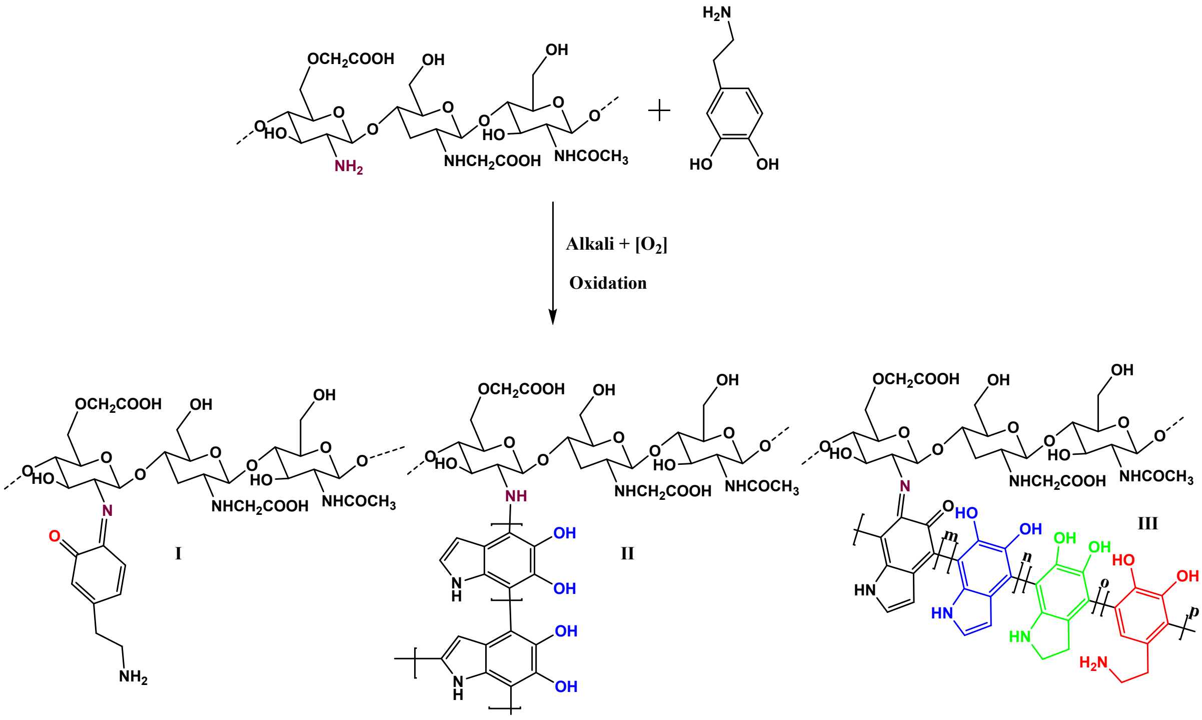

Scheme S1: Schematic representation of possible chemistry of FCMCS-PDA reaction
